# Genome Sequence of *Staphylococcus aureus* PX03, an Acetoin-Producing Strain with a Small-Sized Genome

**DOI:** 10.1128/genomeA.00753-17

**Published:** 2017-09-14

**Authors:** Ge Zhang, Qian Wang, Yulong Su, Shugui Li, Haobao Liu

**Affiliations:** a Key Laboratory of Tobacco Biology and Processing, Tobacco Research Institute, Chinese Academy of Agricultural Sciences, Qingdao, China; b Hainan Provincial Branch of CNTC, Haikou, China; c Yuanjiang Branch of Yunnan Tobacco Monopoly Administration Company, Yuxi, China; d College of Agriculture, Qingdao Agricultural University, Qingdao, China; e Key Laboratory of Tobacco Gene Resources, Tobacco Institute of Chinese Academy of Agricultural Sciences, Qingdao, China

## Abstract

*Staphylococcus aureus* PX03 can produce acetoin efficiently. Here, we present a 2.38-Mb assembly of its genome sequence, which might provide further insights into the molecular mechanism of its acetoin biosynthesis to further improve its biotechnological applications.

## GENOME ANNOUNCEMENT

*Staphylococcus aureus* is a Gram-positive facultative anaerobe which belongs to the *Staphylococcus* family. It is an opportunistic human bacterial pathogen responsible for nosocomial and community-associated infections (1). *Staphylococcus aureus* has been demonstrated to produce acetoin, a flavoring agent widely used in the food, cosmetics, pharmaceutical, and chemical industries (2). Here, we report *Staphylococcus aureus* PX03, a strain which exhibited the potential for use in the industrial production of acetoin. The yield of acetoin reached 41 g/liter with *S. aureus* PX03 in a flask, which was significantly higher than yields for other reported strains. *S. aureus* PX03 was isolated from a soil sample in Hunan Province, China. The size of the genome is about 2.38 Mb, which is smaller than that of all other sequenced *Staphylococcus aureus* genomes (2.65 to 2.95 Mb). The genome data provide useful information for further metabolic reconstruction of the pathways for acetoin production.

The draft genome sequence of *Staphylococcus aureus* strain PX03 was obtained by high-throughput whole-genome shotgun sequencing using Illumina Hiseq 2000 sequencing technology. A 468-bp Illumina paired-end library produced 5,934,226 reads (read length, 90 bp) totaling 502 Mb. The short reads were assembled into genomes using SOAPdenovo, version 2.04 (3), resulting in 17 contigs. The protein-coding open reading frames (ORFs) were predicted using Glimmer version 3.02 (4), tRNA genes were predicted using tRNAscan-SE 1.2 (5), and rRNA genes were predicted using RNAmmer 1.2 (6) and were annotated using the Gene Ontology and Kyoto Encyclopedia of Genes and Genomes (KEGG) databases (7, 8). A comprehensive annotation and comparative genome analysis are under way.

The size of the *S. aureus* PX03 genome is about 2.38 Mb, which is smaller than that of all other sequenced *Staphylococcus* genomes. The GC content is 32.86%. A total of 2,506 coding regions, 57 tRNA genes, and 5 rRNA loci were detected. Over 87% of genes were assigned to specific Clusters of Orthologous Groups (COG) database functional groups.

### Accession number(s).

This whole-genome shotgun project has been deposited at GenBank under the accession no. LFOJ00000000. The version described in this paper is the first version, LFOJ01000000.
